# Distinct patterns of serum hepatitis B core-related antigen during the natural history of chronic hepatitis B

**DOI:** 10.1186/s12876-017-0703-9

**Published:** 2017-12-04

**Authors:** Zhan-Qing Zhang, Xiao-Nan Zhang, Wei Lu, Yan-Bing Wang, Qi-Cheng Weng, Yan-Ling Feng

**Affiliations:** 1 Department of Hepatology, Shanghai Public Health Clinical Center, Fudan University, Caolang Road 2901, Shanghai, 201508 China; 2 Research Unit, Shanghai Public Health Clinical Center, Fudan University, Caolang Road 2901, Shanghai, 201508 China; 3 Shanghai Representative Office, Fujirebio Inc., Shanghai, China; 4 Department of Clinical Pathology, Shanghai Public Health Clinical Center, Fudan University, Shanghai, China

**Keywords:** HBcrAg, HBV, HBsAg

## Abstract

**Background:**

The current clinical practice on chronic hepatitis B (CHB) requires better on-treatment monitoring of viral persistence. Quantified assays for hepatitis B surface antigen (HBsAg) and core-related antigen (HBcrAg) hold promise for further optimization of therapy. Here, we aimed to characterize HBcrAg during the natural course of CHB.

**Methods:**

Four-hundred and forty four treatment naïve CHB patients, who all underwent liver histology examination, were enrolled in this cross-sectional study. Their HBV DNA, HBsAg, HBeAg and HBcrAg titres were quantified and analyzed in the context of four distinct clinical phases. Correlation of HBcrAg and HBsAg with other markers were performed. The relationship between liver and serum antigen levels were also assessed.

**Results:**

HBcrAg, like HBsAg, exhibited high degree of correlation with HBV DNA. However, a more significant linear relationship was found between HBcrAg and HBeAg titre in immune tolerant (IT) and immune clearance (IC) phases, while in HBeAg negative hepatitis (ENH) group, HBV DNA is a major determinant of HBcrAg. Significant difference was observed in liver HBcAg score and HBcrAg level in both IT and IC phases whereas barely significant positive correlations between liver HBsAg score and HBsAg titre was documented.

**Conclusion:**

HBcrAg titre exhibited distinct correlative profile in a phase-specific manner. In addition, its level is well-related to the intrahepatic expression of core antigen. It has a considerable utility in monitoring and refining antiviral therapy.

## Background

The hepatitis B virus continues to be a global public health issue with over 240 million chronically infected individuals worldwide. Chronic hepatitis B virus (CHB) infection is connected with a high risk of developing liver fibrosis, cirrhosis and hepatocellular carcinoma, which results in over 780,000 deaths annually [1]. The natural history of CHB is commonly regarded as consisting of four phases [2]; immune-tolerant (IT), immune-clearance (IC), non/low-replicative (LR), and hepatitis B e antigen negative hepatitis (ENH). These phases have been classified by specific biochemical, serological and virological characteristics, including serum ALT levels, hepatitis B e antigen (HBeAg) serostatus, and hepatitis B virus DNA (HBVDNA) titre.

A deeper understanding of the pathogenesis and natural history of CHB has been facilitated by the improved sensitivity of HBV DNA viral load assays, reliable assays for serum hepatitis B surface antigen (HBsAg) and HBeAg [2, 3] and the development of assays for the detection and measurement of HBV intrahepatic reservoir, covalently closed circular DNA (cccDNA) [4]. As a relatively new serum immunoassay, quantification of hepatitis B core-related antigen (HBcrAg) provided additional virological information regarding the status of chronic HBV infection [5]. The HBcrAg assay detects the sum of hepatitis B core antigen (HBcAg), e antigen (HBeAg) and its related byproduct, 22-kDa precore protein (p22cr) [6]. It has been shown to have close correlation with HBV DNA but exhibit less decline after antiviral therapy [7]. Thus it was proposed as a surrogate marker for HBV persistence [8].

In this study, we aimed to further evaluate the HBcrAg assay in 444 treatment naïve CHB patients spanning all four phases of its natural history, all of whom underwent liver histological examinations. The relationships between HBcrAg and serum, liver markers were analyzed in a phase-specific manner.

## Methods

### Patients

This study included 444 Chinese patients with CHB who were hospitalized at the Shanghai Public Health Clinical Center of Fudan University between January 2012 and September 2015. The diagnosis of all the patients were made according to with the “Guideline on the prevention and treatment for chronic hepatitis B” (2010 version) jointly released by the Chinese Society of Hepatology and the Chinese Society of Infectious Diseases, Chinese Medical Association. The detailed inclusion and exclusion criteria were previously described [9].

Patients were classified into one of four phases of CHB based on HBeAg serostatus, HBV DNA and serum ALT levels. The Immune tolerant (IT) phase was defined as: HBeAg positive, HBV DNA > 20,000 IU/mL, serum ALT ≤ 2 × upper limit of normal (ULN). The Immune clearance (IC) phase was defined as: HBeAg positive, HBV DNA > 20,000 IU/mL, serum ALT > 2 × ULN. The low replicative (LR) group was defined as: HBeAg negative, HBV DNA < 2000 IU/mL and normal serum ALT. The E negative hepatitis (ENH) phase was defined as: HBeAg negative, HBV DNA > 2000 IU/mL, serum ALT >2 × ULN.

### Liver biopsy and pathological diagnosis

The detailed procedures for ultrasound-assisted liver biopsy and pathological evaluations of necro-inflammatory activity and fibrosis were previously described [9]. The pathological diagnosis referred to the Scheuer standard, in which grade is used to describe the intensity of necro-inflammatory activity, and stage is a measure of fibrosis and architectural alteration. The grades include five levels, G0–G4, and the stages include five levels, S0-S4 [10].

For immunohistochemistry (IHC), formalin-fixed paraffin embedded sections were routinely dewaxed and rehydrated. After heat induced antigen retrieval in sodium citrate (pH 6.0) buffer, sections were incubated with the primary monoclonal antibody against HBsAg (clone 1044/341, Novocastra) and rabbit polyclonal anti-HBcAg antibodies (Dako). After washing, the polymer detection system (Polink-1 HRP, GBI Labs) was incubated for 30 min at room temperature and developed with 3, 3′-diaminobezdine (DAB). The expression of liver HBsAg and HBcAg was evaluated by semiquantitative scoring method. The scores include four levels: 0 (no positive cells), 1 (positive cells <25%), 2 (positive cells 25% -49%), 3 (positive cells ≥ 50%).

### Laboratory assays

Measurement of serum HBcrAg was performed using a CLEIA Lumipulse G1200 automated analyzer (Fujirebio Inc., Tokyo, Japan) and the reagents were provided by Fujirebio Inc., lot number: SAX5031 (Japan) [5]. Serum HBsAg and HBeAg were measured using a chemiluminescence microparticle immunoassay Abbott Architect I2000 automated analyzer (Abbott Laboratories, Chicago, IL, USA) [11, 12]. The detailed measurement procedures were previously described [9]. Serum HBV DNA levels were measured using a Bio-Rad Icycler PCR System (Bio-Rad Laboratories, Inc., California, USA), and the PCR kits were obtained from Qiagen Shenzhen Co. Ltd. (China). The linear detection range was 5 × 10^2^ IU/mL to 5× 10^7^ IU/mL.

### Statistical analyses

Statistical analyses were performed using PASW version 18.0 (SPSS Inc., Chicago, Illinois, USA). The Mann-Whitney U test and Kruskall-Wallis ANOVA test were used for non-parametric continuous data. Prearson’s correlation coefficient was used for analyzing the correlation among logarithmized serum HBcrAg, HBsAg, HBeAg and HBV DNA values. Multiple linear regression analysis was employed to evaluate the contribution of HBeAg and HBV DNA to the overall readout of HBcrAg.

## Results

### Basic characteristics of enrolled patients

The basic characteristics of enrolled patients are presented in Table 1. Based on the criteria described above, they were classified into four distinct groups: i.e., IT (*n* = 158), IC (*n* = 133), LR, (*n* = 99) and ENH (*n* = 54). The male-to-female ratios in these four groups were generally comparable (*p* = 0.053). As expected, HBeAg negative patients (LR and ENH group) were older than HBeAg positive patients (IT and IC group, *p* < 0.001). In terms of liver histology, significant lower scores of necro-inflammation and fibrosis were documented in IT and LR groups compared with IC and ENH groups (*p* < 0.001), which agreed well with their phase classification.

**Table 1:** Baseline characteristics of enrolled patients

	Immune Tolerant (*n* = 158)	Immune clearance (*n* = 133)	Low Replicative (*n* = 99)	HBeAg negativ hepatitis (*n* = 54)	*p* value
Age	34 (28–42)	31 (26–39)	42 (32/49)	43 (37–52)	<0.001^a^
Gender M/F	92/66	94/39	58/41	39/15	0.053^b^
ALT IU/L	40.5 (24–58.5)	175 (103–356)	22 (14–30)	160 (112–391)	<0.001^a^
HBV DNA log IU/ml	7.65(6.45–7.70)	7.50 (6.65–7.69)	<2.70 (<2.70–2.73)	5.81 (5.10–6.74)	<0.001^a^
HBsAg log IU/ml	4.43 (3.54–4.76)	3.96 (3.55–4.52)	3.96 (3.56–4.51)	3.28 (2.78–3.69)	<0.001^a^
HBeAg log COI	2.97 (1.93–3.13)	2.80 (2.12–3.08)	Negative	negative	–
Necro-inflammation (G1:G2:G3)	109:23:26	36:50:47	82:10:7	18:17:19	<0.001^a^
Fibrosis: (S1, S2, S3, S4)	84:38:16:20	33:48:20:32	65:13:10:11	13:15:12:14	<0.001^a^
Liver HBsAg (0:1:2:3)	1:29:71:57	3:30:44:56	20:38:25:16	2:13:22:17	<0.001^a^
Liver HBcAg (0:1:2)	78:50:30	85:38:10	96:2:1	49:5:0	<0.001^a^

Data expressed as the median (interquartile range)

^a^Kruskall–Wallis analysis

^b^chi square test

### The level of HBcrAg and its relationship with other markers

**Fig. 1:**
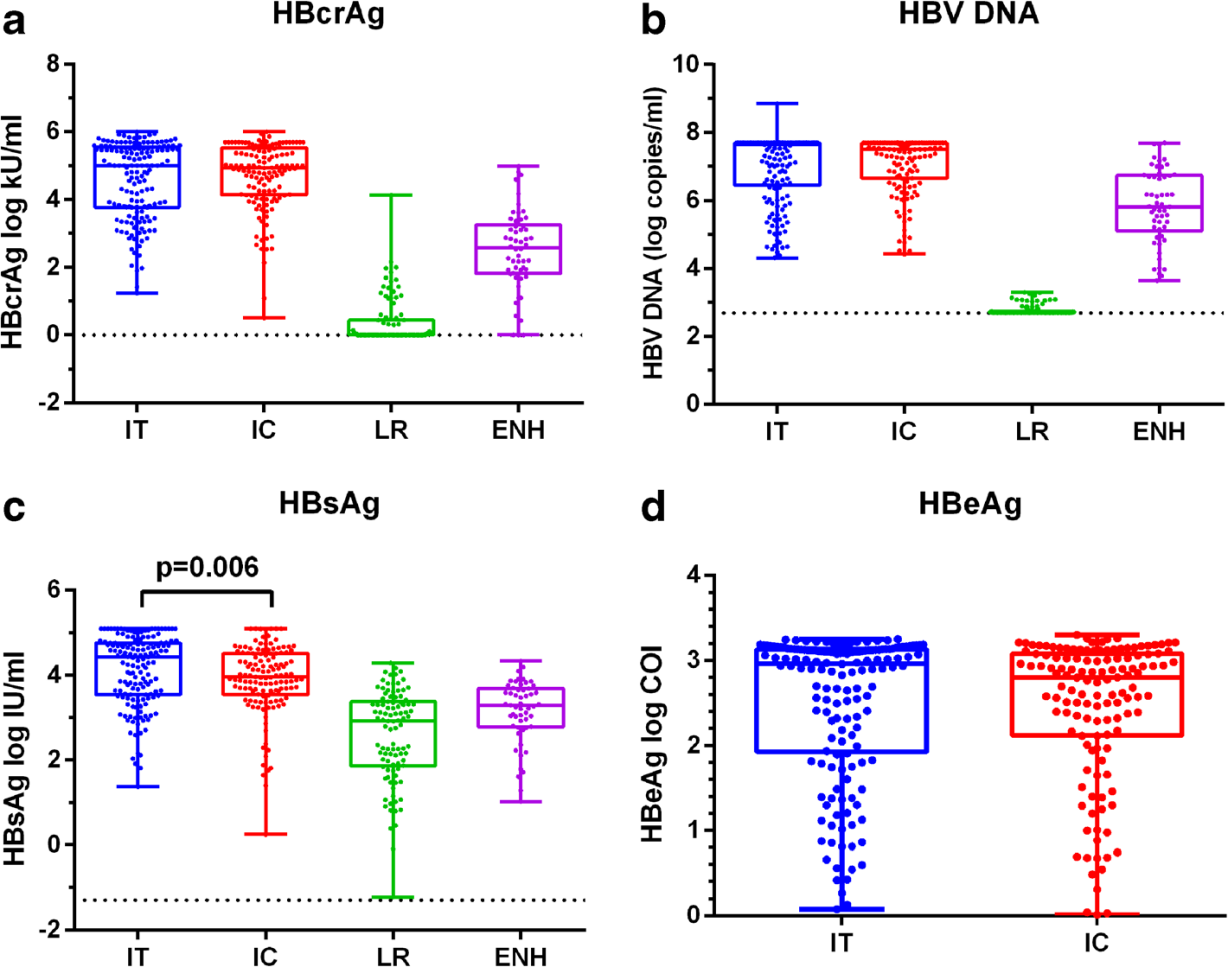
Distribution of HBcrAg (**a**), HBV DNA (**b**), HBsAg (**c**) and HBeAg (**d**) throughout the natural history of CHB. Plots withbox and whiskers combined with dots were drawn in each phase of CHB. The detection limit for HBcrAg and HBV DNA were shown as dashed lines. IT, immune-tolerant; IC, immune-clearance; LR, low-reaplicative; ENH, HBeAg negative hepatitis

The levels of HBcrAg across four phases of diseases were evaluated (Fig. 1a). Highest level of HBcrAg was observed in IT (median 5.00 log kU/ml) and IC (4.94 log kU/ml) whereas lowest level was found in LR group, in which 61 of 99 patients tested negative for HBcrAg. The median level of HBcrAg in ENH group is 2.58 log kU/ml with three tested negative. The general feature of HBcrAg distribution is similar to that of HBV DNA (Fig. 1b) and HBsAg (Fig. 1c). However, the HBsAg titre exhibited less dramatic changes across four phases, and the HBcrAg titre did not show significant difference between IT and IC group as opposed to HBsAg (*p* = 0.006, Mann-Whitney U test). The HBeAg titre between IT and IC group were generally comparable (Fig. 1d).

**Fig. 2:**
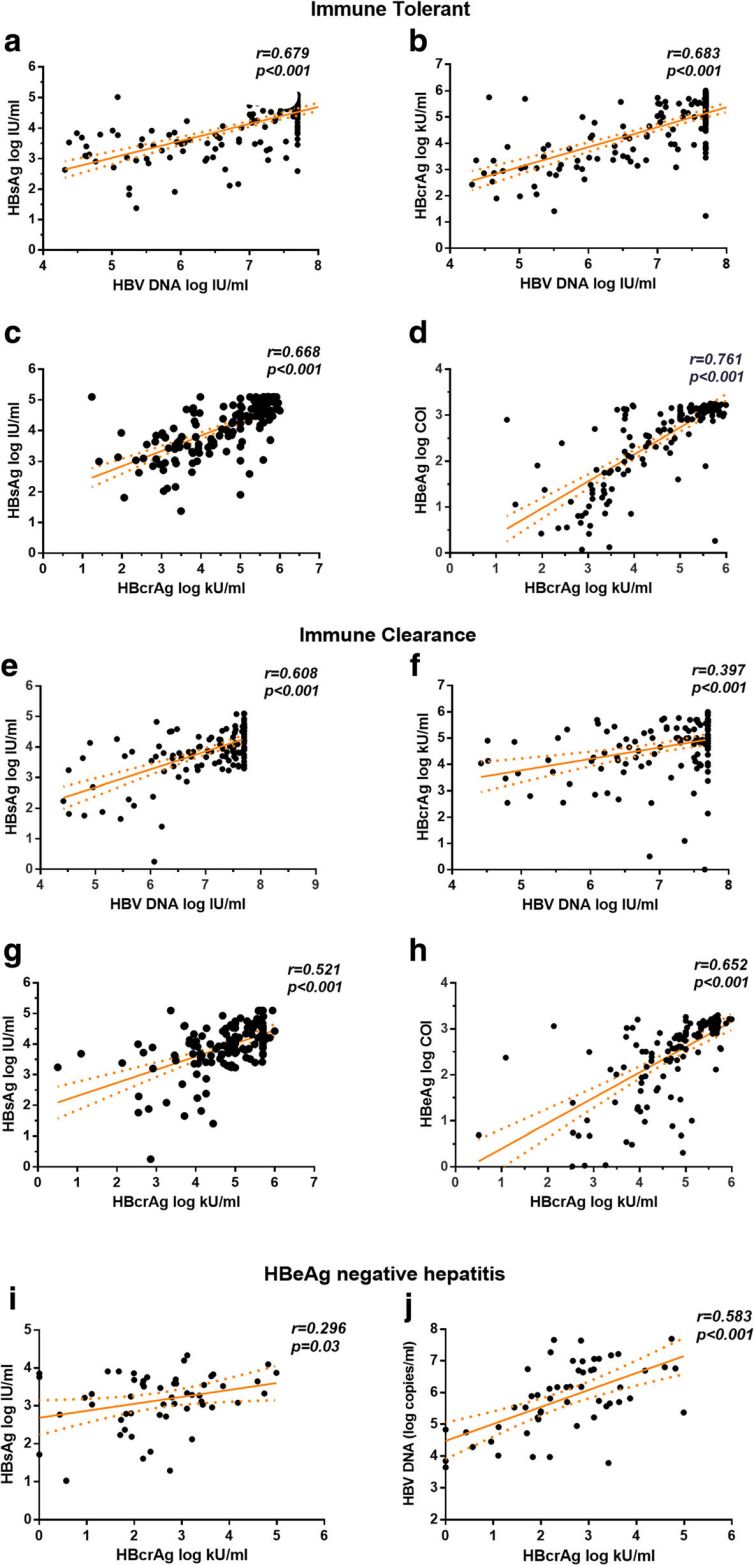
Correlation of major viral markers in immune-tolerant (**a**-**d**), Immune-clearance (**e**-**h**) and HBeAg negative hepatitis (**i**, **j**) groups.Pearson’s correlation coefficient, *p* value and estimate of linear regression curve were shown in each plot

We next evaluated the correlation patterns among levels of major viral antigens and nucleic acid in a phase-specific manner. In agreement with a large body of literature, HBV viral antigen and DNA levels exhibited high degree of correlation in IT and IC phases (Fig. 2a-h). However, we noticed that HBcrAg and HBeAg showed highest correlation coefficient in IT (*r* = 0.761) and IC group (*r* = 0.652) (Fig. 2d, h and Table 2). In ENH group, HBcrAg and HBV DNA showed higher level of correlation (*r* = 0.583, Fig. 2j) compared with HBsAg and HBcrAg (*r* = 0.296, *p* = 0.03, Fig. 2i, Table 2). In LR group, a lack of correlation was found between HBV DNA and HBsAg/HBcrAg (data now shown) mainly because a relatively less sensitive qPCR assay was used (detection limit 500 copies/ml). However, statistically significant correlation was found between HBsAg and HBcrAg titre (*p* < 0.001, *r* = 0.368, Table 2).

**Table 2:** Phase-specific correlation of HBcrAg and HBsAg with various biomarkers

	Immune Tolerant (*n* = 159)		Immune clearance (*n* = 134)		Low Replicative (n = 99)		HBeAg negativ hepatitis (*n* = 54)	
	r	P	r	P	r	P	r	P
HBcrAg								
HBV DNA (log IU/ml)	**0.683**	**<0.001**	**0.397**	**<0.001**	0.023	0.822	**0.583**	**<0.001**
HBeAg (log COI)	**0.761**	**<0.001**	**0.652**	**<0.001**	NA	NA	NA	NA
HBsAg (log IU/ml)	**0.668**	**<0.001**	**0.521**	**<0.001**	**0.368**	**<0.001**	0.296	0.03
ALT IU/L	−0.78	0.330	0.082	0.133	0.06	0.555	0.126	0.365
HBsAg								
HBV DNA (log IU/ml)	**0.679**	**<0.001**	**0.608**	**<0.001**	0.224	0.026	0.380	0.005
HBeAg (log COI)	**0.624**	**<0.001**	**0.456**	**<0.001**	NA	NA	NA	NA
HBcrAg (log IU/ml)	**0.668**	**<0.001**	**0.521**	**<0.001**	**0.368**	**<0.001**	0.296	0.030
ALT IU/L	−0.08	0.271	0.104	0.231	0.015	0.879	−0.092	0.509

The r and *P* values of the correlation analyses were in bold if statistical significance is prominent (*p*<0.001)

### Multiple linear regression analysis of HBcrAg

As the HBcrAg titre is actually the sum of HBcAg, HBeAg and its related byproduct, and the amount of HBcAg can be approximated with HBV viral load, we reasoned that HBcrAg readout can be decomposed into two major elements, i.e., HBV DNA and HBeAg titre. Thus, we performed multiple linear regression of HBcrAg titre using HBV viral load and HBeAg titre as variables (Table 3). Interestingly, we found that in HBeAg positive patients (IT and IC group), HBeAg titre is the major factor for HBcrAg (*r* = 0.751, *p* = 1.99E-11 in IT group, *r* = 0.697, *p* = 3.35E-13 in IC group) whereas HBV DNA level had a minor effect (*r* = 0.262, *p* = 0.004 in IT group, *r* = 0.164, *p* = 0.057 in IC group). In ENH group however, with HBeAg absent, HBV DNA exhibit significant linear relationship with HBcrAg (*p* = 0.635, *p* = 3.68E-6).

**Table 3:** Multiple linear regression analysis of HBcrAg

Clinical Variables	Regression Coefficient	SE of Regression Coefficient	95% CI		*P* value	Multiple Regression Coefficient (R)
			Lower	Upper		
Immune tolerant						**0.776**
logHBV DNA	**0.262**	**0.089**	**0.087**	**0.437**	**0.004**	
logHBeAg	**0.751**	**0.104**	**0.546**	**0.956**	**1.99E-11**	
Immune clearance						**0.664**
logHBV DNA	0.164	0.085	−0.005	0.333	0.057	
LogHBeAg	**0.697**	**0.086**	**0.527**	**0.867**	**3.35E-13**	
E negative hepatitis						**0.583**
LogHBV DNA	**0.635**	**0.123**	**0.389**	**0.881**	**3.68E-6**	

The r and *P* values of the correlation analyses were in bold if statistical significance is prominent (*p*<0.001)

### The relationship between circulating antigens and their intrahepatic status

**Fig. 3:**
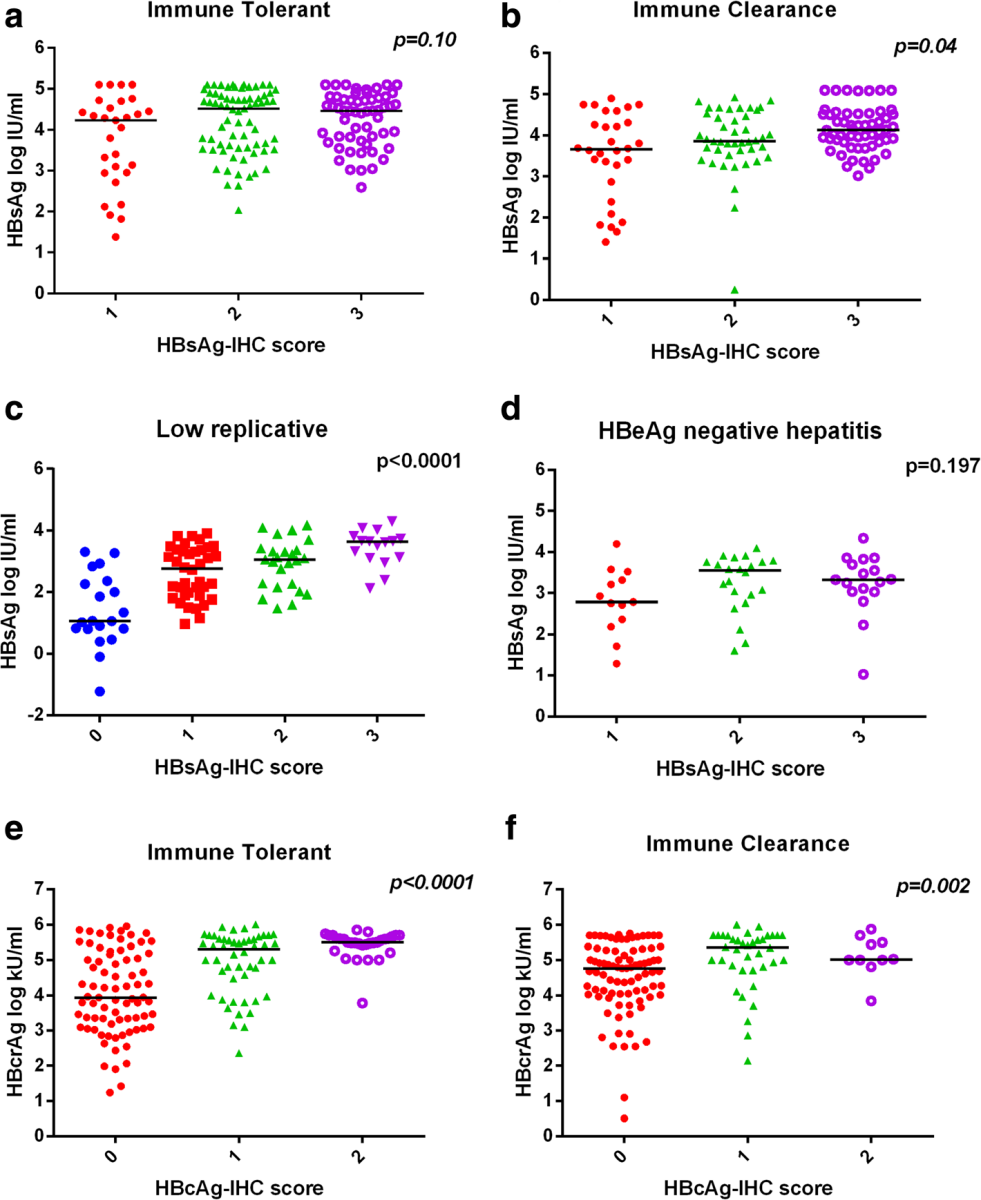
Relationship between circulating antigens and their intrahepatic status. The HBsAg (**a**-**d**) and HBcrAg (**e**, **f**) titres in each phases were grouped according to immunohistochemistry scores of HBsAg and HBcAg respectively and plotted

We then tried to evaluate whether the levels of circulating HBsAg, HBcrAg corresponded to the intrahepatic abundance of their counterparts, i.e., HBsAg and HBcAg. We grouped the IT and IC patients according to their HBsAg or HBcAg immunohistochemistry scores and analyzed their corresponding serum antigens. It was found that there was a barely significant difference in HBsAg titre when grouped with liver HBsAg score in IT (*p* = 0.10, Fig. 3a) and IC group (*p* = 0.04, Fig. 3b). However, a gradual increase in HBsAg titre was found in accordance with higher HBsAg score in liver biopsy of LR group (*p* < 0.0001, Fig. 3c) whereas no difference was found in ENH group (*p* = 0.196, Fig. 3d). In terms of HBcrAg, significant differences were observed in HBcrAg level when grouped with liver HBcAg score in both IT (*p* < 0.0001, Fig. 3e) and IC group (*p* = 0.002, Fig. 3f). In LR and ENH group, due to the very limited cases positive for intrahepatic HBcAg (3 in LR and 5 in ENH group, Table 1), no meaningful statistical analysis was possible.

## Discussion

The hepatitis B virus exploits its limited genome for transcription of various messenger RNAs and pregnomic RNA. In addition, it utilizes alternative translation initiation to express various polypeptide products from a single messenger. HBV encodes two core-related open reading frames (ORFs), i.e., precore and core ORF. The precore ORF express a 25-kDa polypeptide containing an N terminal 19-aa signal peptide, which is cleaved during translocation into the ER lumen [11]. After its subsequent cleavage of its carboxy terminus by furin endopeptidase [12], a 17-kDa mature HBeAg is secreted. In addition, a 22-kDa precore protein (p22cr) was reported, whose N-terminal signal peptide was not cleaved and lacked arginine-rich C terminal domain critical for nucleic acid binding. It was found to be assembled into Dane-like particles but devoid of HBV genome [6]. The core ORF expresses the 21.5-kDa HBcAg which assembles into dimers and form the capsid. In serum of HBV patients, HBV capsids were believed to be encapsidated by HBV surfaces antigens to form Dane particles although naked capsids were reported in hepatoma cell lines [13].

Since HBcAg is encapsidated by viral envelope and high activity of HBcAg neutralizing antibody in the serum, its level cannot be easily quantified. Kimura et al. reported a sensitive enzyme immunoassay for quantifying HBV core-related antigens using monoclonal antibodies reactive to denatured HBcAg and HBeAg. This assay exhibited a detection limit of 4 pg/ml and was insensitive to interfering anti-HBc and anti-HBe antibodies in specimens. Clinical evaluation of the HBcrAg assay found that, although it was highly correlated with HBV viral load, its decline after antiviral therapy was less pronounced compared with HBV DNA [7]. Hence, HBcrAg was proposed to be a marker for viral persistence. Indeed, van Campenhout et al. reported that HBcrAg levels were associated with response to entecavir and Peginterferon add-on therapy in HBeAg positive patients [14]. Furthermore, Honda et al. reported that HBcrAg is related to intra-hepatic HBV replication and development of hepatocellular carcinoma [15].

Previously, in a total of 205 Chinese CHB patients, all of whom underwent liver histology examination, we found that HBcrAg is useful in predicting the necro-inflammation and advanced fibrosis [9]. In this study, we analyzed the relationship between HBcrAg and other viral markers in more detail, based on basic molecular biology of HBV precore and core proteins, in 444 CHB patients spanning all four phases of CHB natural history. It should be noted, that European Association for the Study of Liver recently issued a updated guideline for management of hepatitis B virus [16], in which a new nomenclature is used to describe these four phases, i.e., HBeAg-positive chronic infection, HBeAg-positive chronic hepatitis, HBeAg-negative chronic infection and HBeAg-negative chronic hepatitis. Nevertheless, the criteria for patient classification is essentially unchanged and will not affect the statistical analysis in this study.

We found, by multiple linear regression, that the major variance of HBcrAg can be attributed to HBeAg in IT and IC groups, while in ENH group, HBV DNA is a major determinant of HBcrAg. It should be noted that these linear regression analyses were by no means intended to formulate an accurate quantitative relationship between HBcrAg and HBVDNA/HBeAg. They were utilized to better elucidate the major contributors of HBcrAg in different stages of diseases. Indeed, these results could well explain the less pronounced decline of HBcrAg level during antiviral therapy compared with HBV DNA [7]. However, it should be acknowledged that this analysis still did not fully reflect the real picture since genome-free HBV virions containing HBcAg were reported to exist in high molar ratio to Dane particles [13] and p22cr, which can assemble into Dane-like particles devoid of HBV DNA, also exists [6]. Nevertheless, our analysis revealed that HBcrAg titre exhibited a phase-specific relationship with serum viral biomarkers such as HBeAg, HBsAg and HBV DNA.

Apart from circulating biomarkers, our analysis also looked into the abundance of viral antigens (HBsAg, HBcAg) in different compartments (serum and liver). A significant positive relationship was observed in liver HBcAg-IHC scores and HBcrAg level. Since most of the core antigen staining was found within hepatocyte nuclei [4], it is conceivable that over-production of precore and core antigens would lead to core antigen nuclear transport and accumulation. As to the surface antigen, it was reported that the major intrahepatic S protein is large surface antigen, while circulating S protein was dominated by small surface antigen [17]. These large S protein can even accumulate in high levels which induces ground-glass morphology [18]. It is possible that these differences lead to the lack of correlation between liver and serum surface antigens in IT, IC and ENH groups. Nevertheless, the existence of around 20% HBsAg-IHC negative cases in LR group may be responsible for the statistical significance in this particular phase.

## Conclusions

In conclusion, although several articles have already been published on the features of HBcrAg during the natural course of CHB [19, 20], our analyses provided additional information. First, HBcrAg titre were statistically related to HBeAg and HBV DNA which exhibited a phase-specific dominance pattern. This pattern can be well explained by the basic virology of hepatitis B virus. Second, the histological examinations on all enrolled 444 patients revealed a clear correlation between HBcrAg and intrahepatic HBcAg. Indeed, quantitative assays for core-related antigen has shown its utility in monitoring and refining antiviral therapy. It would be most desirable that novel precision assays could be developed to quantify all major products of precore and core ORF (HBcAg, p22cr, HBeAg etc) which would allow detailed analyses on their dynamics in the natural course of disease and their immune-modulatory roles leading to life-time persistent infection.

## Abbreviations

CHB: Chronic hepatitis B
ENH: HBeAg negative hepatitis
HBcAg: Hepatitis B core antigen
HBcrAg: Hepatitis B core-related antigen
HBeAg: Hepatitis B e antigen
HBsAg: Hepatitis B surface antigen
HBV: Hepatitis B virus
IC: Immune clearance
IT: Immune tolerance
LR: Low replicative
ULN: Upper limit of normal

## References

[1] WHO: Guidelines for the prevention, care and treatment of persons with chronic hepatitis B infection.; 2015. 26225396

[2] Nguyen T, Thompson AJ, Bowden S, Croagh C, Bell S, Desmond PV, Levy M, Locarnini SA, Hepatitis B (2010). Surface antigen levels during the natural history of chronic hepatitis B: a perspective on Asia. J Hepatol.

[3] Thompson AJ, Nguyen T, Iser D, Ayres A, Jackson K, Littlejohn M, Slavin J, Bowden S, Gane EJ, Abbott W (2010). Serum hepatitis B surface antigen and hepatitis B e antigen titers: disease phase influences correlation with viral load and intrahepatic hepatitis B virus markers. Hepatology.

[4] Zhang X, Lu W, Zheng Y, Wang W, Bai L, Chen L, Feng Y, Zhang Z, Yuan Z (2016). In Situ analysis of intrahepatic virological events in chronic hepatitis B virus infection. J Clin Invest.

[5] Kimura T, Rokuhara A, Sakamoto Y, Yagi S, Tanaka E, Kiyosawa K, Maki N (2002). Sensitive enzyme immunoassay for hepatitis B virus core-related antigens and their correlation to virus load. J Clin Microbiol.

[6] Kimura T, Ohno N, Terada N, Rokuhara A, Matsumoto A, Yagi S, Tanaka E, Kiyosawa K, Ohno S, Maki N, Hepatitis B (2005). Virus DNA-negative dane particles lack core protein but contain a 22-kDa precore protein without C-terminal arginine-rich domain. J Biol Chem.

[7] Rokuhara A, Tanaka E, Matsumoto A, Kimura T, Yamaura T, Orii K, Sun X, Yagi S, Maki N, Kiyosawa K (2003). Clinical evaluation of a new enzyme immunoassay for hepatitis B virus core-related antigen; a marker distinct from viral DNA for monitoring lamivudine treatment. J Viral Hepat.

[8] Suzuki F, Miyakoshi H, Kobayashi M, Kumada H (2009). Correlation between serum hepatitis B virus core-related antigen and intrahepatic covalently closed circular DNA in chronic hepatitis B patients. J Med Virol.

[9] Zhang ZQ, Lu W, Wang YB, Weng QC, Zhang ZY, Yang ZQ, Feng YL (2016). Measurement of the hepatitis B core-related antigen is valuable for predicting the pathological status of liver tissues in chronic hepatitis B patients. J Virol Methods.

[10] Goodman ZD (2007). Grading and staging systems for inflammation and fibrosis in chronic liver diseases. J Hepatol.

[11] Garcia PD, JH O, Rutter WJ, Walter P (1988). Targeting of the hepatitis B virus precore protein to the endoplasmic reticulum membrane: after signal peptide cleavage translocation can be aborted and the product released into the cytoplasm. J Cell Biol.

[12] Ito K, Kim KH, Lok AS, Tong S (2009). Characterization of genotype-specific carboxyl-terminal cleavage sites of hepatitis B virus e antigen precursor and identification of furin as the candidate enzyme. J Virol.

[13] Ning X, Nguyen D, Mentzer L, Adams C, Lee H, Ashley R, Hafenstein S, Hu J (2011). Secretion of genome-free hepatitis B virus--single strand blocking model for virion morphogenesis of para-retrovirus. PLoS Pathog.

[14] van Campenhout MJ, Brouwer WP, van Oord GW, Xie Q, Zhang Q, Zhang N, Guo S, Tabak F, Streinu-Cercel A, Wang J, et al. Hepatitis B core-related antigen levels are associated with response to entecavir and Peginterferon add-on therapy in Hbeag-positive chronic hepatitis B patients. Clin Microbiol Infect. 2016;22(6):571.e5–9. 10.1016/j.cmi.2016.02.002 26898481

[15] Honda M, Shirasaki T, Terashima T, Kawaguchi K, Nakamura M, Oishi N, Wang X, Shimakami T, Okada H, Arai K (2016). Hepatitis B virus (HBV) Core-related antigen during Nucleos(t)ide analog therapy is related to intra-hepatic HBV replication and development of hepatocellular carcinoma. J Infect Dis.

[16] European Association for the Study of the Liver (2017). Electronic address eee, European Association for the Study of the L: EASL 2017 clinical practice guidelines on the management of hepatitis B virus infection. J Hepatol.

[17] Gerken G, Manns M, Gerlich WH, Hess G, Meyer zum Buschenfelde KH (1989). Immune blot analysis of viral surface proteins in serum and liver of patients with chronic hepatitis B virus infection. J Med Virol.

[18] Hadziyannis S, Gerber MA, Vissoulis C, Popper H, Cytoplasmic h B (1973). Antigen in "ground-glass" hepatocytes of carriers. Arch Pathol.

[19] Maasoumy B, Wiegand SB, Jaroszewicz J, Bremer B, Lehmann P, Deterding K, Taranta A, Manns MP, Wedemeyer H, Glebe D, et al. Hepatitis B core-related antigen (HBcrAg) levels in the natural history of hepatitis B virus infection in a large European cohort predominantly infected with genotypes A and D. Clin Microbiol Infect. 2015;21(6) 606 e601–610 10.1016/j.cmi.2015.02.010 25700889

[20] Seto WK, Wong DK, Fung J, Huang FY, Liu KS, Lai CL, Yuen MF, Linearized h B (2014). Surface antigen and hepatitis B core-related antigen in the natural history of chronic hepatitis B. Clin Microbiol Infect.

